# Evolution, multifunctionality, and agricultural potential of insect microbiomes and the holobiont concept

**DOI:** 10.1093/ismejo/wrag137

**Published:** 2026-05-27

**Authors:** Wei Zhang, Ioannis Eleftherianos, Amr Mohamed, Guy Smagghe, George Joseph Chakkalakkal, Rasha Al-Akeel, Umut Toprak, Gianluca Tettamanti, Nemat Keyhani, David Renault

**Affiliations:** State Key Laboratory of Green Pesticide, Guizhou University, Huaxi District, Guiyang 550025, China; School of Biological Sciences, Institute for Global Food Security, Queen’s University Belfast, Belfast, Northern Ireland BT9 5DL, United Kingdom; Department of Entomology, Faculty of Science, Cairo University, Giza 12613, Egypt; Institute of Entomology, Guizhou University, Guiyang 550025, China; Department of Biology, Vrije Universiteit Brussel (VUB), Brussels 1050, Belgium; School of Biological Sciences, Institute for Global Food Security, Queen’s University Belfast, Belfast, Northern Ireland BT9 5DL, United Kingdom; Department of Zoology, Faculty of Science, King Saud University, Riyadh 11451, Saudi Arabia; Ankara University, Faculty of Agriculture, Department of Plant Protection, Ankara 06110, Türkiye; Department of Biotechnology and Life Sciences, University of Insubria, via J.H. Dunant, 3, Varese 21100, Italy; BAT Center-Interuniversity Center for Studies on Bioinspired Agro-Environmental Technology, University of Napoli Federico II, Piazza Carlo di Borbone, 1, Portici 80055, Italy; Department of Biological Sciences, University of Illinois, Chicago, IL 60607, United States of America; Université de Rennes 1, UMR CNRS 6553 Ecobio, 263 Avenue du Gal Leclerc, CS 74205, Rennes 35042, France

**Keywords:** insect gut microbiota, hologenome theory, microbial detoxification, symbiotic mutualism, genome editing, sustainable agriculture

## Abstract

Insect-associated microbiomes, as co-evolved members of the holobiont, play pivotal roles in host physiology, ecological resilience, and evolutionary innovation. This review synthesizes recent advances in understanding microbial symbionts’ contributions to metabolic adaptation, insecticide detoxification, and immune modulation. Framed within hologenome theory—which posits host-microbe assemblages as units of natural selection—we explore co-evolutionary dynamics driving mutualistic specialization and adaptive plasticity. Cutting-edge tools like genome editing and metagenomics reveal how gut microbiota mediate cross-kingdom interactions, insecticide resistance, and reproductive fitness. Intriguingly, microbial symbionts can enhance host resistance through detoxification while sensitizing hosts to specific toxins, highlighting context-dependent trade-offs. Targeted manipulation of microbial consortia—via detoxification disruption or symbiont engineering—offers new avenues for sustainable pest control, though ecological risks demand rigorous biosafety protocols. A paradigm shift toward holobiont-centered models promises unified strategies for sustainable agriculture and biodiversity conservation in the Anthropocene.

## Graphical abstract

**Figure:**
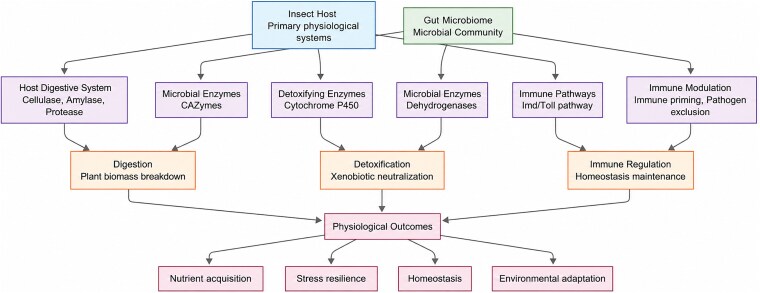

## Introduction

As Earth’s most species-rich animal group, insects have dominated terrestrial and aquatic ecosystems for over 480 million years, radiating into more than one million described species [1]. Their remarkable evolutionary success stems not merely from intrinsic adaptations but from sophisticated alliances with microbial symbionts—bacteria, fungi, and archaea—that have co-evolved to become indispensable partners [2]. Microbial consortia drive metabolic innovation in nutrient-poor environments [3, 4], detoxify plant secondary metabolites [5], and xenobiotics [6, 7]. In some instances, they have the potential to transform environmental challenges into evolutionary opportunities [8]. Far from passive commensals, these microorganisms’ function as integral components of the insect holobiont. The holobiont is a unified biological entity where the combined genetic repertoire of host and microbiome evolve as a single adaptive unit [9–11], and giving birth to the hologenome concept.

For superorganisms, hologenomes facilitate evolutionary responses when facing environmental challenges allowing insects to overcome genomic constraints through microbial outsourcing [12]. For instance, the 200-million-year association between aphids and *Buchnera* bacteria has transformed these microbial partners into dedicated amino acid factories, enabling hosts to thrive on nutritionally deficient phloem sap [13]. Similarly, leaf-cutter ants’ agricultural symbiosis with *Leucoagaricus* fungi, dating back 50 million years, represents a sophisticated system of biomass conversion where fungal cytoplasmic contents are repurposed as ant nutrients [14]. The evolutionary significance of these partnerships is further exemplified by ambrosia beetles. These insects have indeed developed specialized mycangia to cultivate fungal symbionts [15].

The Anthropocene era presents unprecedented challenges to these delicate associations, in addition to pushing insects to acquire unique microorganisms that can degrade xenobiotics. Global insecticide application exceeding 3.7 million tons annually has driven resistance evolution in over 600 pest species [16, 17], with gut microbiota playing an increasingly recognized role in detoxification. Recent studies demonstrated that *Pseudomonas* species degrade pyrethroids [18], *Burkholderia* neutralizes organophosphates [19, 20], and *Serratia* reprograms host gene expression to enhance chemical resistance [21]. Paradoxically, certain *Enterobacter* strains amplify *Bacillus thuringiensis* (*Bt*) toxicity in lepidopterans by suppressing host immunity and accelerating protoxin activation, ultimately increasing *Plutella xylostella* mortality [22]. This dual capacity for both detoxification and toxicity enhancement underscores the microbiome’s complex role in insect survival strategies. Variations in the composition of microbiota which improve resistance to insecticide may have consequences for organismal biology (e.g. reproduction, development, longevity, energy acquisition) and fundamental physiological processes. Microorganisms have several critical roles in: (a) immune modulation through ROS regulation and NF-κB signaling [23–25], (b) nutrient and metabolite exchange within the holobiont [26–28], (c) pheromone production and reproductive manipulation [29–31], and (d) microbiota-derived acetate remodeling *Drosophila* lipid metabolism [32].

Here, our review synthesizes contemporary understanding of insect-microbe systems through ecological, evolutionary, and molecular lenses. We argue that the insect microbiome should be viewed as a “phenotypic plasticity engine”. Specifically, the microbiome is a dynamic genomic resource that enables holobionts to track environmental change, resist and adapt to chemical stressors, and exploit other ecological niches that would have not been colonized by the host genome alone. In this review, we develop our assumption across three biological functional axes—detoxification, immune modulation, and reproductive fitness—and place the holobiont as the fundamental unit of selection in insect adaptation to anthropogenic pressures. Following a brief presentation of the roles played by the symbiotic bacteria and fungi, we then consider the insect gut microbiota and host interactions, their roles in the regulation of host metabolism, immunity, reproduction, and resistance to pesticides. Finally, we evaluate CRISPR-based symbiont engineering and holobiont-informed approaches for sustainable pest management. In an era of ecological crisis, we conclude by re-emphasizing the critical importance of recognizing insects as complex holobionts rather than isolated organisms. In doing so, we pave the way to future research directions that may unlock new strategies for biodiversity conservation and agricultural sustainability through targeted manipulation of their microbial partnerships. This review focuses on terrestrial insects spanning major pest and model taxa (Diptera, Lepidoptera, Hemiptera, Coleoptera, and Hymenoptera), covers literature primarily from 2012 to 2025, and addresses both obligate and facultative bacterial and fungal symbionts, with an emphasis on functional outcomes relevant to fundamental biology and applied pest management.

### Roles and dynamic nature of the symbiotic gut microbes

**Figure 1:**
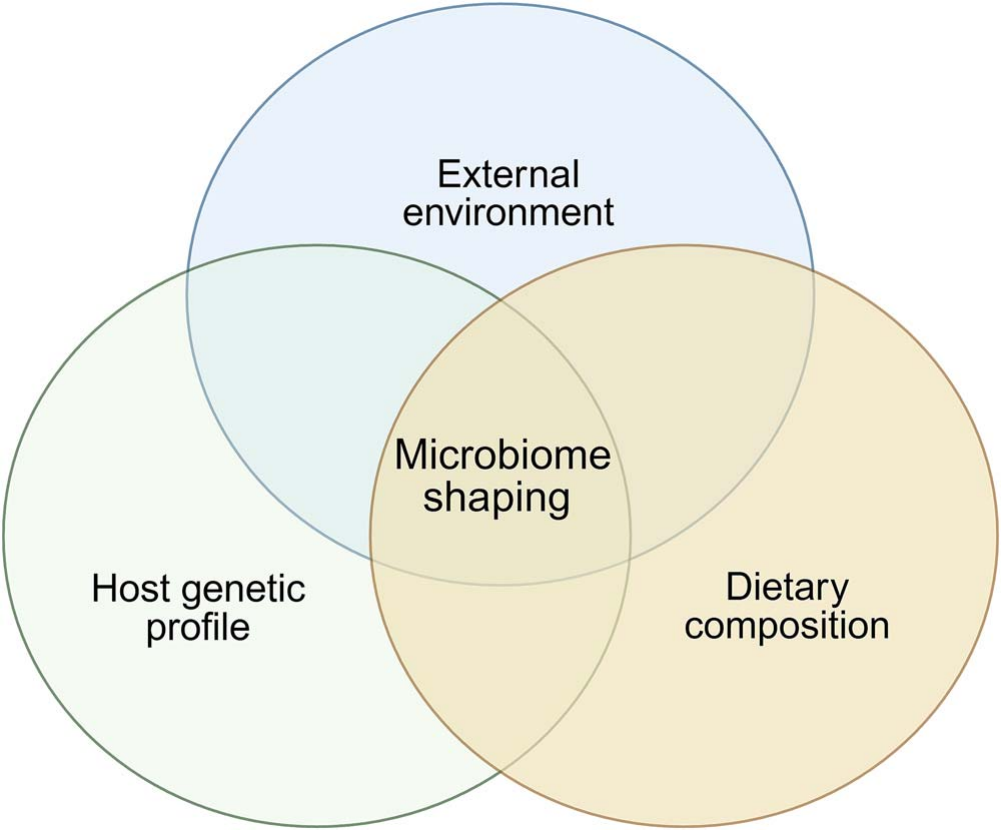
Factors governing the diversity and functional aspect of the host microbiome. The Venn diagram represents how host genetics, diet, and environmental parameters collectively shape the host microbiome. The overlap among these elements highlights the complex interplay of intrinsic and extrinsic factors that together determine the microbiome composition.

The insect gut microorganisms exhibit remarkable plasticity, undergoing dynamic restructuring in response to host developmental stage, dietary composition, environmental fluctuations, and genetic factors (Fig. 1) [33–35]. This microbial flexibility represents a critical adaptive mechanism, enabling insects to maintain physiological homeostasis across diverse ecological niches [36].

Diet serves as a primary driver of microbiome composition, particularly in herbivorous insect species. In *Spodoptera frugiperda* (fall armyworm), different host plant species (e.g. corn vs wheat or field-grown vs laboratory diets) drive significant shifts in dominant bacterial phyla and genera, with *Enterococcus* and *Enterobacter* commonly abundant when feeding on plant hosts, whereas *Proteobacteria* (*Acinetobacter* and *Rhodococcus*) expand under insecticide exposure [37, 38]. These shifts appear to modulate capacities for detoxification and xenobiotic metabolism (e.g. sublethal insecticide exposure increases taxa with known metabolic versatility). In other words, plant host characteristics, particularly nitrogen availability and resulting metabolic landscape, shape microbial communities that co-evolve or respond plastically to metabolize both plant defensive compounds and synthetic insecticides.

In *Spodoptera litura*, for example, high-nitrogen diets (both by fertilization of maize plants and in artificial diets) significantly promote *Enterococcus* populations in the gut [39]. Two strains, *Enterococcus mundtii* and *Enterococcus casseliflavus*, isolated from such high-N guts, when reintroduced, enhance larval tolerance to methomyl; conversely, when antibiotics are included in high-N diets, methomyl sensitivity increases again—strong evidence of a causal nutritional–microbial synergy. This suggests that agricultural practices (e.g. fertilization) may indirectly select for higher insecticide tolerance via effects on gut microbiota.

Nutritional–microbial synergies extend to diverse systems, including *Riptortus pedestris* and *Cletus punctiger*, where *Burkholderia* symbionts are acquired from the environment, colonize midgut crypts, degrade insecticides like fenitrothion into non-toxic metabolites, and even use the byproducts as carbon sources [40, 41]. In *C. punctiger*, experimental infection with fenitrothion-degrading *Burkholderia* strains yields higher survival under insecticide exposure, and the degrading activity is maintained in the midgut, suggesting the functional significance of microbial detoxification.

Dietary-induced microbial changes directly influence digestive efficiency and nutrient assimilation. A striking example emerges from *Rhynchophorus ferrugineus* (red palm weevil), where gut microbiota modulate hemolymph nutrient profiles—including triglycerides and glucose levels—thereby regulating larval development and growth rates [42].

Because diet (especially nitrogen) clearly shapes both microbial community composition and functional capacity in these systems, future work should (i) test more precisely the fitness costs or trade-offs associated with microbiome-mediated tolerance, (ii) examine how plant secondary metabolites interact with nitrogen to influence microbiome selection, and (iii) clarify whether these shifts are stable across generations and in field conditions.

**Figure 2:**
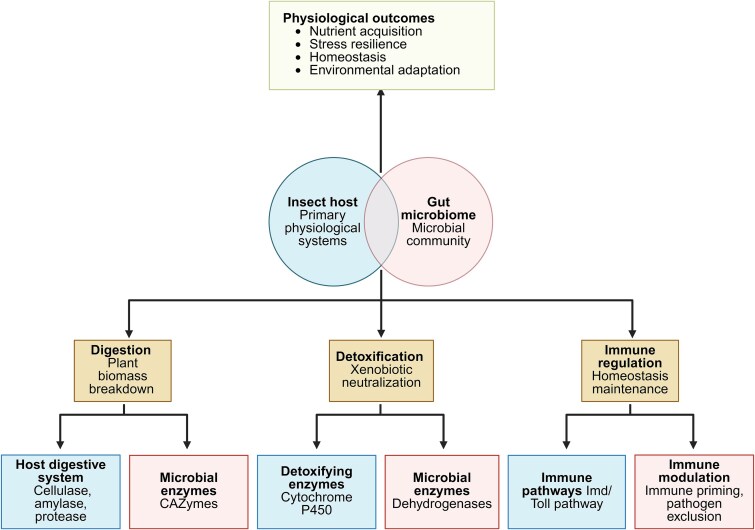
Host-microbiome association modulating digestion, detoxification, and immune system regulation in insects. This flowchart depicts the interactions between the insect host and its gut microbiome across primary physiological functions, such as digestion, detoxification, and immune regulation. The tight coupling between the host and the microbiome highlights their integrated role in maintaining physiological homeostasis and facilitating adaptation to dietary and environmental challenges.

Beyond nutritional roles, gut microbiota actively participate in immune system priming and pathogen defense (Fig. 2). Members of the Burkholderiaceae family can penetrate gut epithelia, triggering systemic immune responses through antimicrobial peptide production and phagocyte activation [43]. A particularly elegant example of microbiome-mediated immunity occurs in *Spodoptera littoralis* (cotton leafworm), where *E. mundtii* produces the selective antimicrobial mundticin KS. This compound specifically targets invasive pathogens while sparing commensal bacteria, thereby maintaining microbial equilibrium while enhancing host defense capabilities [44]. Such sophisticated microbial governance of host immunity highlights the evolutionary refinement of insect-microbe partnerships.

The microbiome exhibits distinct temporal dynamics across an insect’s life history. In hemimetabolous species (which lack a pupal stage), recent evidence shows that microbial community turnover is more strongly shaped by maternal care and social interactions than by the physical process of molting. According to recent work, in the European earwig (*Forficula auricularia*), microbiome diversity (alpha and beta) shifts non-linearly across developmental stages—but these shifts are *not* associated with moulting events [45]. Furthermore, the presence of the mother early in life alters microbiome composition not only in juveniles during family life, but also later in adults—months after maternal contact ends, indicating lasting effects of early microbial acquisition via maternal care [45].

Other systems provide parallel examples include: in *Nicrophorus vespilloides* (a burying beetle), larvae receiving full parental care (pre-hatch treatment of carcass + feeding of larvae) are colonized predominantly by maternal-derived bacteria; those without care get more environmental bacteria [46]. In holometabolous polyphagous flies, as larvae mature and post-metamorphosis adults emerge, microbiome diversity often expands in larval stages and contracts in adult stages, suggesting strong developmental stage effects beyond simple molting [47].

The importance of microorganisms in seasonal adaptations are particularly evident in *Loxostege sticticalis* (beet webworm moth), where gut microbiota undergo significant compositional and functional shifts between nondiapause and prediapause states, facilitating host adaptation to changing environmental conditions [48]. Similarly, *Halyomorpha halys* (brown marmorated stink bug) exhibits seasonally dynamic microbiomes, with *Candidatus Pantoea carbekii* dominating year-round populations whereas *Commensalibacter* emerges as a summer-specific symbiont, reflecting resource-driven microbial succession [49]. We have now evidence that the host genotype interacts with environmental factors to shape microbial communities, as demonstrated in *Hermetia illucens* (black soldier fly), where genetic background, age, and diet collectively influence gut microbiota composition and host performance metrics [50]. This genotype-environment-microbiome interplay underscores the complex determinants of microbial assembly in insects.

### Fungal symbionts in insect microbiomes: Architects of mutualistic adaptation which have replaced ancient bacterial symbionts

**Figure 3:**
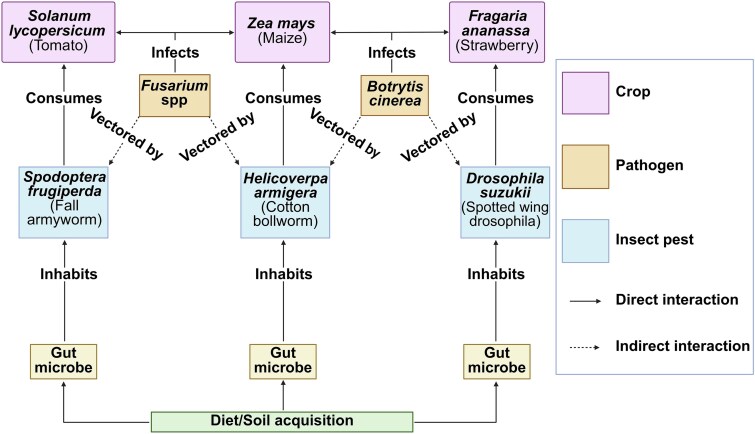
Ecological multitrophic interactions involving insect hosts, gut microbiota, crops, and pathogens. Ecological network illustrating multitrophic interactions among insect hosts, their gut microbiota, crops, and associated pathogens. The diagram highlights both direct interactions, such as microbial acquisition from diet and soil, microbial activity within the insect gut, and insect–crop feeding relationships; and indirect interactions, including crop susceptibility shaped by shared microbial pools and insect-mediated pathogen transmission. Together, these connections underscore the complexity of multitrophic dynamics within agricultural systems.

Fungal symbionts represent cornerstone components of insect microbiomes, having evolved intricate mutualisms that fundamentally shape host evolutionary trajectories and ecological success (Fig. 3) [51, 52]. As such, fungal symbionts emerged as co-architects of insect evolutionary success, and these associations transcend transient interactions, frequently developing into obligate partnerships where fungi serve as: (i) metabolic specialists, (ii) biochemical defense systems, and (iii) ecological niche expanders [53]. Through these multifaceted roles, fungal symbionts have become indispensable partners in insect adaptation and diversification.

The evolutionary hallmark of insect-fungal partnerships lies in the host’s outsourcing of critical metabolic functions. Three exemplary systems demonstrate this principle: (i) Detoxification Specialists: Cigarette beetles rely on *Symbiotaphrina* yeasts to neutralize toxic coumarins in seeds, enabling exploitation of otherwise inaccessible resources [54]. (ii) Biomass Converters: Ambrosia beetles (*Xyleborus* spp.) maintain *Raffaelea* fungi in specialized mycangia, utilizing fungal lignocellulases to degrade plant cell walls—a capability absent in the beetle genome [55]. (iii) Nutrient Provisioners: Termites (Isoptera) cultivate *Termitomyces* fungal gardens that transform refractory plant polymers into digestible nutrients, facilitating their wood-based diets [56]. These symbioses often drive reductive genome evolution in fungal partners, eliminating redundant metabolic pathways and cementing their dependence on host insects [57].

Fungal symbionts frequently serve as biochemical shields against environmental threats. For instance, leaf-cutter ants (*Atta* spp.) leverage *Leucoagaricus gongylophorus* for dual purposes: nutrient acquisition and pathogen suppression through antimicrobial metabolite production [58, 59]. Bark beetles (*Dendroctonus* spp.) employ *Grosmannia clavigera* to detoxify conifer terpenes, overcoming formidable plant chemical defenses [60].

The persistence of host–microbe partnerships over evolutionary time depends on sophisticated transmission strategies. For example, in the coffee berry borer (*Hypothenemus hampei*), females carry spores of *Fusarium solani* in cuticular structures (pronotal asperites), which act like primitive mycangia, ensuring that offspring acquire the fungus from the start [61]. In a different system, there is apparent phylogenetic congruence between *Drosophila* species and their yeast symbionts (*Hanseniaspora*), implying that the host and symbiont lineages have diversified in parallel [62].

Insect-fungal partnerships frequently produce ecosystem-level impacts. For example, burying beetles (*Nicrophorus* spp.) utilize *Photorhabdus* fungi to preserve carrion resources via microbial competition suppression [63, 64]. The yeast-like symbiont of *Nilaparvata lugens* mediates replacement of ancient bacterial partners through genomic specialization [65]. These interactions underscore how mycangia - the diverse, independently evolved fungal storage organs in ambrosia beetles - represent remarkable examples of morphological adaptation to symbiosis.

The hologenome framework reveals how fungal partnerships drive adaptive radiation. Gall midges (Cecidomyiidae) leverage fungal symbionts to overcome host plant constraints, facilitating niche expansion [66]. This “genetic outsourcing” enables insects to transcend genomic limitations, creating evolutionary trajectories shaped by combined host-fungal capabilities.

### Insect gut microbiota and host interactions

The insect gut, resident microbiota perform indispensable functions that extend far beyond basic nutrition. Microbial symbionts contribute to the breakdown of refractory plant polymers through the production of cellulases, proteases, and pectinases (Fig. 2) [67], while also synthesizing essential nutrients such as B vitamins and amino acids that compensate for nitrogen-limited diets [2]. Equally critical is their role in detoxifying both plant secondary metabolites and synthetic pesticides, enabling host survival in chemically challenging environments (Fig. 2) [63, 64]. In return, the host provides a stable, nutrient-rich habitat that sustains microbial populations while benefiting from their metabolic activities—a reciprocal relationship that profoundly influences insect development, immune function, and reproductive success [68]. The physiological importance of this symbiosis is underscored by the developmental impairment and reduced fitness observed in germ-free insects [69, 70], as well as by the microbiota’s newly recognized role in enhancing host tolerance to environmental stressors including temperature fluctuations and osmotic challenges [71].

Anatomically, the insect gut comprises three functionally distinct regions, each with specialized structural adaptations. The foregut and hindgut, derived from ectodermal tissue, are lined by protective chitinous cuticle [72]. In contrast, the midgut—the primary site of digestion and nutrient absorption—lacks a cuticular lining but is safeguarded by a dynamic peritrophic matrix (PM). This intricate structure, composed of chitin fibrils embedded in a proteinaceous matrix, forms a semi-permeable barrier around the food bolus with multiple protective functions. Beyond providing mechanical protection to the midgut epithelium, the PM serves as a selective filter that regulates microbial transit while preventing pathogen invasion [73, 74]. Recent research has revealed that PM formation by the midgut epithelium is actively modulated by the gut microbiota through immune signaling pathways such as IMD, with bacterial peptidoglycan acting as a key stimulus [75]. The PM’s critical role in vector competence has become increasingly apparent, as its structural integrity directly influences pathogen transmission efficiency in disease-carrying insects [76, 77]. This defensive function is exploited by certain pathogens, including malaria parasites that secrete chitinases to degrade the PM barrier [78], highlighting the evolutionary arms race between insect hosts and their microbial challengers.

### Microbial regulation of insect physiology and metabolism

The gut microbiota drive several insect key physiological processes (Fig. 2). In *D. melanogaster* for instance, commensal bacteria play an indispensable role in host growth and development by activating insulin/TOR signaling pathways through acetic acid production. This microbial metabolite serves dual functions: (i) as a signaling molecule that orchestrates lipid metabolic remodeling and (ii) as a direct nutritional substrate for the host [79]. The physiological consequences of microbial deprivation are striking, with germ-free drosophila flies exhibiting developmental retardation and metabolic dysfunction that can be fully reversed through microbial reintroduction. Riboflavin-producing gut bacteria additionally maintain mitochondrial enzyme function in female *Drosophila*, with riboflavin deficiency in germ-free individuals leading to impaired mitochondrial activity that is correctable through either bacterial recolonization or direct vitamin supplementation [80].

Nutritional symbioses often exhibit remarkable specialization across insect taxa. For instance, the silkworm *Bombyx mori* harbors a gut microbial community uniquely adapted to its monophagous diet of mulberry leaves. In this insect species, methylobacteriaceae members degrade aromatic compounds and participate in phytohormone synthesis, although cyanobacterial and chloroplast-derived taxa contribute to cellulose breakdown and vitamin production [81]. Concurrently, *Lactobacillus* and *Weissella* species ferment dietary sugars to generate short-chain fatty acids that serve as important energy sources [48]. In phloem-feeding aphids (*Acyrthosiphon pisum*), *Buchnera aphidicola* provides all essential amino acids and vitamins absent from the nutritionally imbalanced phloem diet [82], thus making nutritional symbiosis conditional to aphid survival. Several other evolutionary adaptations are found in a large of insect models: leafcutter ants cultivate *Leucoagaricus* fungi for plant biomass conversion [83], bed bugs (*Cimex* spp.) rely on *Wolbachia* for B vitamin synthesis [84], and tsetse flies depend on *Wigglesworthia glossinidia* for blood meal digestion and reproductive nutrition [85]. Intriguingly, *Wigglesworthia* populations appear regulated according to long-term host nutritional requirements rather than immediate dietary status [86], suggesting sophisticated evolutionary tuning of these symbiotic relationships.

To counteract phytochemical defenses, gut symbionts has the potential to neutralize or transform these toxic compounds. When *Trichoplusia ni* (cabbage looper) feeds on *Solanum lycopersicum* (tomato), its gut microbiome shifts to favor *Agrobacterium* and *Rhizobium* strains capable of alkaloid detoxification [87]. Similar adaptations occur in *Hyles euphorbiae* and *Brithys crini*, where *Enterococcus* species confer alkaloid tolerance [88], and in *Hypothenemus hampei* (coffee berry borer), where *Pseudomonas fulva* degrades caffeine to maintain host fitness [89]. The cruciferous plant defense system presents a different challenge through glucosinolates, which *T. ni* overcomes via gut bacteria (*Shinella, Terribacillus*, and *Propionibacterium*) that break down these compounds [87], whereas *Pantoea* sp. in *Psylliodes chrysocephala* restores isothiocyanate degradation capacity [64].

Flavonoid detoxification showcases particularly elegant microbial adaptations. *Dendroctonus valens* (pine beetle) relies on *Novosphingobium* to neutralize naringenin [90], whereas *B. mori* gut microbiota glycosylate prenylated isoflavones from mulberry, converting them to fewer toxic derivatives [91]. Oxalate-degrading symbionts like *Ishikawaella capsulata* in *Megacopta punctatissima* [92] and *Bacillus subtilis* in *D. melanogaster* protect their hosts from calcium precipitation disorders [93].

The microbial arms race extends to other defensive compounds: Acinetobacter in *Curculio chinensis* degrades saponins [94], specialized symbionts in *Lymantria dispar* break down tannins [95], and bark beetle-associated *Serratia, Pseudomonas*, and *Rahnella* metabolize α-pinene [96–98]. Even phenolic glycosides in unripe fruits are detoxified by *Erwinia dacicola* in *Bactrocera oleae* [99], whereas honeybee gut bacteria (*Bifidobacterium* and *Gilliamella*) neutralize amygdalin in almond nectar [100].

Microbial symbionts confer critical abiotic stress tolerance to their insect hosts. The whitefly *Bemisia tabaci* demonstrates enhanced thermal resistance through *Rickettsia*-mediated upregulation of stress-response genes [101]. Temperature adaptation mechanisms vary across systems: in *Culex pipiens* mosquitoes, yeast symbionts are essential for cold tolerance [102], whereas in *Bactrocera dorsalis*, the gut bacterium *Klebsiella michiganensis* modulates arginine and proline metabolism to enhance cold resilience [103]. These findings collectively underscore the gut microbiome’s pivotal role as a mediator of insect environmental adaptation, with important implications for understanding pest ecology under changing climatic conditions.

### Immune modulation by gut symbionts

The gut microbiome maintains a delicate equilibrium between pathogen defense and symbiotic tolerance through sophisticated molecular dialogues. In *D. melanogaster*, commensal bacteria engage in continuous crosstalk with host immune pathways, where Gram-negative symbionts activate the Imd-Relish (NF-κB) pathway through peptidoglycan recognition by PGRP-LC/LE receptors [25]. This basal immune activity serves dual physiological functions: (i) antimicrobial peptide (AMP) production maintains microbial community balance, while (ii) concurrent stimulation of intestinal stem cell proliferation ensures epithelial renewal and PM maintenance. The essential nature of this microbial priming is evident in germ-free flies and Imd-pathway mutants, which exhibit impaired gut epithelium regeneration [104]. This system operates as a self-regulating circuit, where commensal-induced NF-κB activation simultaneously prevents microbial overgrowth while avoiding destructive inflammation [105].

A parallel defensive mechanism involves lactobacilli-stimulated Duox/Nox enzymes that generate controlled levels of reactive oxygen species (ROS) in gut epithelium [106]. These ROS molecules act as antimicrobial agents that regulate microbial populations and as signaling molecules that maintain tissue homeostasis.

Recent research has revealed the complex trade-offs inherent in host-microbe immune interactions. Although *Lactobacillus plantarum*–derived lactate activates protective Nox-mediated ROS production, excessive activation can lead to oxidative stress that paradoxically reduces host lifespan [107]. Similarly, the Imd pathway modulates the growth-promoting effects of *Acetobacter pomorum*, with immune-deficient mutants showing accelerated larval development [108]. These findings illuminate the evolutionary balancing act between harnessing microbial benefits and controlling their potential costs.

The existing knowledge suggests that principles of microbiome-mediated immunity are conserved across insect taxa. In *Anopheles gambiae*, native gut microbiota provide basal protection against *Plasmodium* infection, with antibiotic-treated mosquitoes showing increased susceptibility to malaria parasites [109]. Specific symbionts like *Serratia marcescens* enhance this protection through both immune pathway activation and direct parasite inhibition [110]. Extreme examples of microbial-mediated defense occur in fungus-farming ants, where cuticular *Acinetobacter* bacteria produce antibiotics that protect fungal gardens from competitors [111], and in monarch butterflies, where gut microbes influence both immune gene expression and flight muscle development crucial for migration [112]. These diverse systems collectively demonstrate how insect microbiomes engage conserved immune pathways (Imd, Toll, JAK/STAT, DUOX/ROS) to orchestrate tripartite interactions between host, symbionts, and pathogens.

### Impact on growth, development, and reproduction

Gut microbiota are drivers of several insect life history traits, and regulate growth trajectories, developmental programs, and reproductive strategies. In *R. pedestris* (bean bug), the selective enrichment of *Burkholderia* symbionts within specialized midgut compartments significantly enhances reproductive output, demonstrating how host-mediated microbial recruitment and interbacterial dynamics can directly impact fitness [113]. Similarly, the gut microbiome of *Bactrocera tryoni* (Queensland fruit fly) orchestrates a suite of physiological processes including larval and adult body mass regulation, feeding behavior modulation, lipid storage dynamics, and female fecundity, with microbiota-dependent sexual dimorphism evident in carbohydrate utilization patterns [114]. The functional importance of these microbial partners is strikingly demonstrated by the complete restoration of normal physiology upon microbial reintroduction in axenic individuals.

Recent discoveries have unveiled mechanisms through which gut microbes directly modulate insect reproductive biology. Male *B. dorsalis* (olive fruit fly) harbor *Bacillus* spp. in their rectal glands that biochemically transform glucose and threonine into volatile pyrazines (2,3,5-trimethylpyrazine and 2,3,5,6-tetramethylpyrazine), which function as potent sex pheromones to attract conspecific females [115]. This microbial pheromone biosynthesis illustrates the roles of symbionts in mediating intersexual communication. Parallel phenomena occur in *Drosophila suzukii* (spotted wing drosophila), where *Enterobacter* sp. AA26 supplementation enhances male mating success while optimizing developmental parameters in mass-rearing conditions [116], and in wild *Ceratitis capitata* (Mediterranean fruit fly) populations, where gut microbiome composition influences cuticular hydrocarbon profiles and consequent mate selection outcomes [117].

The developmental consequences of microbiome disruption underscore the ecological importance of these symbiotic relationships. Antibiotic treatment in *S. frugiperda* results in reduced feeding activity, growth retardation, and prolonged developmental duration, highlighting the microbiota’s critical role in nutrient processing and energy allocation [118]. Holometabolous insects like *Onthophagus taurus* (taurus scarab beetle) exhibit particularly sophisticated solutions to the challenges of microbial maintenance during metamorphosis. Despite the absence of functional digestive systems during egg and pupal stages, these beetles preserve core microbial communities through ingenious host-constructed refugia, including fecal microenvironments and pupal chambers, ensuring intergenerational transmission of symbionts essential for developmental fitness [119]. Such conserved microbial inheritance patterns challenge conventional views of microbiome plasticity during radical ontogenetic transformations, suggesting strong selection for symbiont-mediated developmental stability in certain insect lineages.

### Microbial detoxification as a shield against insecticides and modulation of insect resistance

Insect gut microbiota have emerged as key players in conferring resistance to synthetic insecticides through diverse detoxification mechanisms (Fig. 2). These symbiotic microorganisms demonstrate remarkable metabolic versatility, capable of neutralizing a wide spectrum of chemical classes including organophosphates, pyrethroids, organochlorines, and newer compounds such as avermectins and neonicotinoids [120–123]. It is important to note, however, that the magnitude of microbiome-driven resistance varies considerably across systems: while symbiont-mediated detoxification can account for the majority of resistance in specific host-insecticide combinations (e.g. up to 100-fold survival enhancement in *Burkholderia*-colonized *R. pedestris* exposed to fenitrothion; [40]), in other contexts the microbiome plays a minor or negligible role relative to host genomic mechanisms such as target-site mutations or constitutive P450 upregulation [124]. These caveats underscore the need for case-by-case evaluation rather than blanket assertions about microbiome-driven resistance. The significance of this microbial detoxification capacity is particularly evident in hemipteran systems, where *Burkholderia* strains in *R. pedestris* (bean bug), *C. punctiger* (rice stink bug), and *Cavelerius saccharivorus* (oriental chinch bug) enzymatically degrade fenitrothion into non-toxic metabolites that subsequently serve as carbon sources for both microbiota and host [41, 125].

Dipterans similarly benefit from microbial detoxification pathways. In *B. dorsalis* (oriental fruit fly), *Citrobacter freundii* metabolizes trichlorphon into less toxic derivatives [126], whereas the honeybee (*Apis mellifera*) gut microbiome enhances pesticide resistance through dual mechanisms: upregulation of host cytochrome P450 enzymes in conventional bees compared to their germ-free counterparts [127], and direct degradation of neonicotinoids like clothianidin by specific gut bacteria (*Serratia, Rahnella, Pantoea*, and *Enterobacter*) [128].

Lepidopteran systems reveal additional dimensions of microbial-mediated detoxification. *S. frugiperda* utilizes *Arthrobacter nicotinovorans* and *Pseudomonas stutzeri* to degrade various insecticides including deltamethrin and chlorpyrifos ethyl [129]. Pesticide-exposed larvae selectively enrich microbial strains with specific degradative capacities absent in naive populations [130], demonstrating the adaptive potential of gut microbiota under chemical selection pressure. Even urban pests like *Blatta orientalis* (Oriental cockroach) benefit from *Pseudomonas*-mediated pyrethroid degradation, converting α-cypermethrin into non-toxic metabolites [131].

The contribution of symbiotic microbes to insecticide resistance extends well beyond direct detoxification, encompassing sophisticated regulation of host physiological and molecular processes. Through modulation of metabolic pathways, immune responses, and gene expression networks—particularly those involving detoxification enzymes—gut microbiota significantly enhance their hosts’ capacity to withstand chemical stressors.

In *Anopheles stephensi*, resistance to the organophosphate temephos is mediated in part by symbiotic *Pseudomonas, Aeromonas*, and *Exiguobacterium*, which stimulate the activity of key detoxification enzymes including glutathione S-transferases (GSTs) and esterases [132]. This microbial-mediated enzymatic boost provides mosquitoes with enhanced protection against chemical stress. A parallel phenomenon occurs in *Aedes aegypti* larvae, where exposure to naled and propoxur induces gut microbial community shifts that correlate with upregulated expression of host cytochrome P450 enzymes. These microbial influences appear to generalize across multiple insecticide classes, as evidenced by similar microbiota-dependent resistance mechanisms against temephos and deltamethrin in *A. aegypti* [133].

The complexity of microbe-mediated resistance is particularly evident in *N. lugens* (brown planthopper), where sublethal imidacloprid exposure drives *Wolbachia* proliferation and consequent upregulation of host detoxification genes [134]. This dynamic interaction demonstrates how microbial symbionts can amplify host resistance phenotypes in a context-dependent manner. Similarly, *Serratia oryzae* in *Aedes albopictus* enhances deltamethrin resistance through coordinated upregulation of detoxification pathways [135], illustrating the sophisticated interplay between microbial symbionts and host genetic machinery.

The above findings collectively reveal a paradigm where gut microbiota serve as both direct detoxifiers and regulators of host resistance mechanisms. By orchestrating complex changes in gene expression and metabolic activity, symbiotic microbes position themselves as key players in resistance to synthetic insecticides.

### Microbial contributions to insecticide toxicity enhancement

Given that microbial symbionts often confer insecticide resistance, emerging evidence also reveals their paradoxical role in exacerbating chemical toxicity, adding complex layers to host-microbe-insecticide interactions. This phenomenon is particularly pronounced in insects exposed to *Bt* toxins, where gut microbiota can potentiate pathogenicity through multiple mechanisms.

In *Plagiodera versicolora* (leaf beetle), resident gut bacteria synergize with Cry3Bb toxin by inducing intestinal epithelium damage and microbial dysbiosis, facilitating bacterial translocation that amplifies the insecticidal effects [136]. Similarly, *Enterococcus* species in *Cnaphalocrocis suppressalis* (rice borer) serve as critical mediators of *Bt* susceptibility, with antibiotic-mediated depletion of these bacteria conferring significant protection against *Bt* toxicity [137]. The diamondback moth (*P. xylostella*) demonstrates an even more severe outcome, where *Bt* protoxin-induced gut microbiota shifts can lead to lethal sepsis as bacteria invade the hemocoel through toxin-compromised epithelial barriers [138].

Beyond *Bt* toxins, microbial symbionts similarly modulate sensitivity to synthetic insecticides through diverse pathways. The presence of *Serratia symbiotica* in *A. pisum* enhances vulnerability to both neonicotinoids (imidacloprid) and organophosphates (chlorpyrifos) [139], whereas *Rickettsia* infection in *B. tabaci* (whitefly) potentiates toxicity across multiple insecticide classes [140]. These findings reveal an underappreciated dimension of insect-microbe-chemical interactions, where certain microbial taxa can transform from protective symbionts to liability factors under insecticide exposure.

The dual capacity of gut microbiota to either mitigate or amplify insecticide effects highlights the context-dependent nature of these tripartite interactions. The microbial mediation of toxicity pathways suggests that microbiome profiling could serve as a valuable tool for assessing pest vulnerability and optimizing insecticide deployment strategies.

### Methodological advances in microbiome research

The field of insect microbiome research has undergone a paradigm shift with the development of cutting-edge technologies that bridge the gap between descriptive observations and mechanistic understanding. Three complementary methodological frontiers are driving this transformation, enabling unprecedented resolution in studying host–microbe interactions.

Metagenomics has revolutionized our capacity to decode microbial communities, moving beyond taxonomic profiling to functional characterization. High-throughput sequencing approaches have identified specialized metabolic pathways in diverse insect systems, from terpene-degradation genes in bark beetle-associated bacteria [96] to lignocellulose-processing systems in wood-feeding beetles [141]. Recent technological refinements, including single-cell amplified genomics, have revealed intricate functional networks in cockroach hindguts, identifying key polysaccharide-degrading taxa (Bacteroides, Dysgonomonas, Parabacteroides) alongside sulfate-reducing bacteria and methanogenic archaea [142]. Meta-transcriptomic analyses of *Bactrocera minax* larvae have further demonstrated how microbial phenol-degradation pathways support host adaptation to toxic plant compounds [143].

Gnotobiotic model systems have emerged as powerful tools for causal inference in microbiome studies. The *D. melanogaster* model has been particularly transformative, with axenic rearing and controlled microbial inoculation enabling precise dissection of symbiont functions. Recent work has employed these systems to demonstrate how *Lactobacillus* strains influence intestinal stem cell proliferation via ROS signaling [106]. These controlled systems have also revealed how specific microbial consortia can rescue developmental defects in germ-free insects, providing direct evidence for microbial contributions to host nutrition and immune development.

Live imaging technologies have opened new windows into dynamic host–microbe interactions. The *Drosophila* imaging window technique allows continuous confocal microscopy of gut epithelia and microbiota in behaving flies [144], whereas the Bellymount system enables noninvasive observation of internal organs and microbial populations [145]. Recent refinements like Bellymount-pulsed tracking [146] combine prolonged imaging with physiological relevance through intermittent feeding periods. When integrated with fluorescent reporter systems and advanced microscopy (two-photon, light-sheet), these approaches are revealing real-time microbial colonization patterns, host immune responses, and metabolic exchanges at cellular resolution.

The convergence of these technological advances—spanning omics, gnotobiology, and live imaging—has transformed insect microbiome research into a rigorous experimental science. This multidisciplinary toolkit provides mechanistic insights that were previously inaccessible, from molecular-level understanding of microbe-host signaling to ecosystem-scale consequences of these interactions. As these methods continue to evolve, they promise to unravel the full complexity of insect-microbe symbioses and their ecological and evolutionary implications.

### Emerging applications in pest management: new avenues opened by CRISPR technologies

One particularly promising direction involves the genetic engineering of symbiotic microbes to serve as precision biological tools. Pioneering work in honeybees has demonstrated this potential through modification of the core gut symbiont *Snodgrassella alvi* to produce double-stranded RNA (dsRNA), effectively harnessing the host’s RNA interference (RNAi) pathway for targeted gene knockdown [147]. These engineered symbionts stably colonize the bee gut [148], enabling sustained suppression of viral pathogens and enhanced host survival. Field-level RNAi applications targeting *Varroa destructor* mites have also demonstrated promising efficacy in reducing infestation levels [149], further underscoring the potential of RNAi-based technologies in pollinator protection.

Recent methodological breakthroughs are expanding our capacity to manipulate insect-associated microbes. CRISPR-free genome engineering techniques permit stable chromosomal modifications in key bee symbionts like *Snodgrassella alvi* and *Bartonella apis* through efficient homologous recombination [150]. This advance facilitates both functional studies and development of probiotic interventions. Furthermore, engineered *S. alvi* strains capable of sensing specific inducer molecules (e.g. isopropyl β-D-1-thiogalactopyranoside, IPTG) and responding with fluorescent reporter expression have been developed, creating powerful tools for noninvasive monitoring of host-microbe dynamics through fecal sampling [151, 152] and offering potential platforms for enhancing pollinator health through microbiome engineering [153].

CRISPR/Cas9 systems have also significantly advanced our ability to study and manipulate gut symbionts. In *A. aegypti*, CRISPR-mediated knockout of the *ompA* gene in gut bacteria revealed distinct insights into biofilm formation and microbial persistence [154]. More ambitiously, the paratransgenesis approach leverages CRISPR to engineer native symbionts that express anti-pathogen effectors—including antimicrobial peptides, RNAi constructs, and insecticidal proteins—which can then spread through target populations [26, 155]. Recent innovations have extended this strategy to diverse systems, from tsetse flies [156] to agricultural pests [157], demonstrating its broad applicability.

Environmental applications of microbial engineering are also gaining traction. Probiotic formulations containing insect-pathogenic bacteria or attractant-degrading microbes show promise for area-wide pest suppression. Although distinct from gut-targeted approaches, these strategies share the common goal of manipulating insect-microbe interactions for control purposes. The remarkable success of *Wolbachia*-based dengue control programs exemplifies the transformative potential of such approaches [158].

The integration of CRISPR-based editing, synthetic biology, and microbiome manipulation is expanding the toolbox for biologically grounded pest control. Engineered symbionts capable of delivering effector molecules or disrupting key physiological pathways have shown promising outcomes in controlled experiments. Likewise, microbiome-based enhancements of pollinator health are being explored as ecologically sustainable interventions. Because these tools are still in early stages of development, emerging data suggest their potential to complement or replace certain chemical insecticides with greater precision and lower off-target risks.

### Strategic applications of microbiota-based pest control

Microbiome modulation can significantly enhance pest vulnerability to conventional control measures. Recent conceptual advances have further highlighted how symbionts contribute to insecticide resistance by modulating host detoxification gene expression and metabolic pathways, particularly via cytochrome P450s, GSTs, and esterases [124, 159]. In *S. frugiperda*, deliberate alteration of gut microbial communities increased sensitivity to insecticides through downregulation of detoxification genes [160]. Moreover, the colonization of plants by *Trichoderma afroharzianum*, a widely used biocontrol agent, negatively affects the development and survival of *S. littoralis* by altering the gut microbiota and its nutritional support to the host [161]. Yeast-based RNAi delivery platforms have also shown promise: engineered *Saccharomyces cerevisiae* expressing dsRNA targeting the *Shaker* gene in *D. suzukii* induced species-specific lethality via neural disruption and behavioral impairment [162], although earlier work targeting γ-tubulin demonstrated compromised larval viability and reproductive capacity [163]. Microbial inoculants designed to interfere with detoxification pathways have also proven effective at overcoming resistance mechanisms in various pest species [164, 165].

When integrated with existing IPM frameworks, microbiota-based strategies offer several advantages over conventional pesticides, including reduced environmental persistence and increased target specificity. However, these approaches necessitate rigorous biosafety evaluation, particularly for genetically modified applications. Key considerations include horizontal gene transfer risks between engineered microbes and native microbiota; potential trophic cascade effects through food web interactions; long-term environmental persistence of introduced microbial strains; evolutionary adaptation in target pest populations. Comprehensive risk assessment protocols must address these concerns through controlled field trials and molecular surveillance [166].

### Future directions: microbiome-driven innovations in pest management

The deepening understanding of insect-microbiome interactions is ushering in a transformative phase for pest management, with emerging technologies poised to revolutionize control strategies. Building upon current knowledge of microbial roles in insecticide resistance, several cutting-edge research directions offer particular promise for developing next-generation solutions. Rather than cataloguing incremental advances, we identify three grand challenges that we believe could redefine the field over the coming decade, each requiring bold interdisciplinary integration to address.

Epigenetic regulation mediated by microbial symbionts represents an advanced frontier for resistance management. **Grand Challenge 1: Developing predictive models of holobiont resilience under climate change and chemical stress.** A fundamental unresolved question is whether—and when—holobiont-level selection can be predicted from the properties of its constituent partners. Addressing this challenge requires integrating multi-omics datasets (metagenomics, meta-transcriptomics, metabolomics) with environmental variables (temperature, insecticide pressure, host plant chemistry) into machine-learning frameworks capable of forecasting resistance emergence at landscape scales. Symbiont-mediated epigenetic mechanisms offer one particularly promising predictive lever: Growing evidence suggests that symbionts like *Wolbachia* can induce heritable changes in host gene expression through DNA methylation [167], whereas microbial influence on long non-coding RNAs may regulate detoxification enzymes such as glutathione S-transferase in *P. xylostella* [168]. These findings open possibilities for epigenetic interventions that could disrupt resistance mechanisms while avoiding direct genetic modification of pest populations.

The development of predictive ecological models represents another critical avenue. **Grand Challenge 2: Engineering synthetic microbial consortia for targeted, durable pest suppression.** Moving beyond single-strain paratransgenesis, the design of cooperative microbial communities optimized for ecological stability, host specificity, and multi-target action represents the next frontier in biological control. Achieving this will require advances in synthetic ecology (predicting consortium stability under field conditions), chassis selection (identifying symbiont strains with robust colonization and safe horizontal-gene-transfer profiles), and regulatory frameworks for deliberate environmental release. Given the demonstrated effects of temperature, altitude, and precipitation on microbiome composition and function [135], integrating environmental data with microbial profiles could enable forecasting of resistance hotspots. Such models would support geographically tailored management strategies that account for regional variations in microbiome-mediated resistance dynamics.

Technological convergence is accelerating innovation in this field. **Grand Challenge 3: Establishing a holobiont-centered regulatory and ecological safety framework for microbiome-based interventions.** As engineered symbionts and microbiome-disrupting agents approach field deployment, the field urgently needs standardized protocols for assessing off-target effects on non-pest holobionts, pollinator health, and soil microbiome integrity. This challenge is inherently interdisciplinary, requiring collaboration among microbial ecologists, regulatory scientists, evolutionary biologists, and agricultural stakeholders. CRISPR-based editing of symbiont genomes offers precise tools to disrupt detoxification pathways, while engineered microbial consortia show promise for in situ degradation of pesticide residues [169]. Diagnostic advances like loop-mediated isothermal amplification assays are enabling real-time monitoring of resistance-associated microbes in field settings. These technologies collectively support the development of precision interventions that target specific resistance mechanisms while minimizing ecological disruption.

A particularly promising approach involves engineering synthetic microbial communities designed for cooperative pesticide degradation. When properly optimized for ecological stability and biosafety, such consortia could provide sustainable, long-term resistance management by simultaneously addressing multiple detoxification pathways. This strategy would complement existing biological controls because it reduces reliance on conventional insecticides.

Future research should also explore the broader physiological impacts of microbiome manipulation, including effects on thermal tolerance and pathogen resistance. By understanding how microbes contribute to overall pest fitness under diverse stress conditions, researchers can develop more holistic management approaches that target multiple vulnerabilities simultaneously.

The path forward requires interdisciplinary integration of omics technologies, synthetic biology, and ecological modeling. This convergence will enable the design of adaptive, microbiome-based management systems that balance effective pest control with environmental sustainability. As these technologies mature, they promise to transform agricultural practices and at the same time support biodiversity conservation and long-term food security - addressing some of the most pressing challenges in modern agriculture.

## Data Availability

Data sharing not applicable to this article as no datasets were generated or analyzed during the current study.

## Abbreviations

AMP, antimicrobial peptide

Bt, Bacillus thuringiensis

CRISPR, clustered regularly interspaced short palindromic repeats

dsRNA, double-stranded RNA

GST, glutathione S-transferase

IMD, immune deficiency (pathway)

IPTG, isopropyl β-D-1-thiogalactopyranoside

IPM, integrated pest management

LAMP, loop-mediated isothermal amplification

NF-κB, nuclear factor kappa B

P450, cytochrome P450 monooxygenase

PM, peritrophic matrix

RNAi, RNA interference

ROS, reactive oxygen species

TOR, target of rapamycin.
